# Variability in the Universality of Electron Ionization Mass Spectrometry Response to Oxygenates in Complex Environmental Mixtures

**DOI:** 10.1021/acs.analchem.5c04153

**Published:** 2025-12-08

**Authors:** Purushottam Kumar, Chenyang Bi, Brian Lerner, John Jayne, Manjula R. Canagaratna, Gabriel Isaacman-VanWertz

**Affiliations:** † Department of Civil and Environmental Engineering, 1757 Virginia Tech, Blacksburg, Virginia VA-24061, United States; ‡ 53777 Aerodyne Research Inc, Billerica, Massachusetts MA-01821, United States

## Abstract

Electron ionization mass spectrometry (EIMS) has been widely used to measure complex atmospheric and environmental mixtures. The sensitivity of analytes in EIMS is typically obtained through a multipoint calibration curve using commercially available standards, but many analytes of interest do not have pure compounds available, and many samples contain a large fraction of unidentified compounds. To address these limitations in quantification, those analytes are often calibrated using a small number of standards with chemical similarities (e.g., shared functional groups). However, little systematic data are available on the variability of EIMS response factors to environmental analytes. In this work, we compare the EIMS response with a flame ionization detector (FID), a near-universal detector for organics, to quantify the variability in EIMS sensitivity to organics in a complex atmospheric mixture, particularly secondary organic aerosols (SOA). Particle-phase oxidation products from four precursor–oxidant pairs were produced, sampled, and analyzed by gas chromatography using a Thermal Desorption Aerosol Gas Chromatograph (TAG) with simultaneous detection by both FID and EIMS. The signals of FID and EIMS are highly correlated across the chromatogram, and peak areas of individual chromatographic peaks measured by both detectors are also closely correlated. The sensitivity of EIMS varies on average by ∼21% relative to FID for peak-by-peak comparison of oxygenated organic compounds within a complex mixture, suggesting that as long as some information is available about an unknown sample to categorize the analytes in the sample, an EIMS can be treated as a universal detector calibrated using chemically similar analytes.

## Graphical abstract

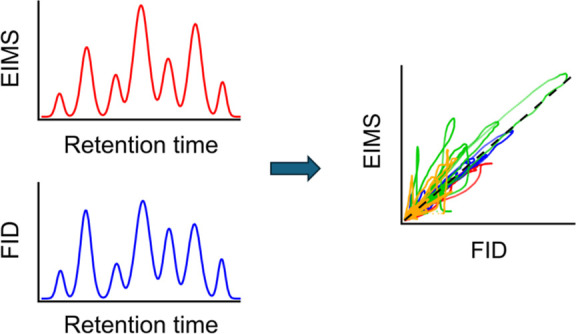

## Introduction

Organic compounds are widely present in environmental samples, which has a significant impact on the global environment and human health.^1^ Consequently, it is important to understand the fate of these organics. However, such efforts have been hindered by the complexity of environmental samples which often contain thousands of different organic compounds. Many of the compounds in environmental samples, particularly oxygenated organics formed through chemical and biological degradation, are not available as authentic standards or easily synthesized. For example, atmospheric samples contain compounds formed through complex photochemical oxidation reactions that generate thousands or tens of thousands of oxygenated products^2,3^ for which no synthesized or commercially available standards are available. The complexity of samples consequently represents a significant challenge in analytical chemistry across a wide range of applications,^4^ and improved methods for quantifying and characterizing these samples are necessary.

Chemical analysis of environmental mixtures is often performed by gas chromatography (GC), which separates components by their physicochemical properties (often volatility), coupled to a flame ionization detector (FID) or a mass spectrometer (MS). An FID measures the ion produced by combustion of an analyte, providing a total concentration of all components eluting at a retention time with a predictable and nearly universal response per unit carbon mass. For oxygenated organics, the average response per unit carbon mass is a function of its chemical functionality, and the sensitivity can be estimated within approximately 20% uncertainty from the oxygen-to-carbon ratio (O/C) of individual analytes.^5^ This detection technique therefore represents an excellent option for quantifying unknown analytes, but suffers from a lack of chemical resolution. To provide substantially improved chemical resolution, many analyses rely on mass spectrometry. The most widely used method for environmental analyses is electron ionization mass spectrometry (EIMS), typically coupled with a GC, owing to its ability to detect nearly all common organic compounds and the existence of mass spectral libraries against which analyte spectral fragmentation patterns can be matched for identification. A “gold standard” approach to determining the sensitivity of analytes in an EIMS is to build multipoint calibration curves using commercially available chemical standards. Unfortunately, this method has limited use for environmental samples due to the lack of available pure compounds due to their chemical or thermal lability.^6^ Instead, the sensitivity of detecting gas-phase molecules by electron ionization can be theoretically determined by the ionization cross-section as a function of electron energy.^7^ Historically, many attempts have been made to predict EIMS sensitivity through experimental measurements^8^ or model estimation^9–11^ of ionization cross-section while other studies tried using empirical approaches to correlate relative EIMS sensitivity with molecular parameters.^12–15^ Unfortunately, those efforts are limited in developing an effective and generalizable method to predict EIMS sensitivities. Even if successful, this approach would have limited utility for environmental samples because a large fraction of analytes do not exist in mass spectral libraries and cannot be identified (∼90% of in atmospheric samples^16,17^ ) stymieing theoretical estimation of their ionization cross-section as well as synthesis of authentic standards.

Additional insights into ionization efficiency in atmospheric applications come from the aerosol community, where instruments such as the Aerosol Mass Spectrometer (AMS) and the Aerosol Chemical Speciation Monitor (ACSM), both developed by Aerodyne Research Inc., utilize electron ionization. Initial estimates of electron impact ionization cross sections in AMS suggested a general scaling with the number of electrons in a molecule. However, differences in the exact scaling factor are observed between inorganic and organic species.^18,19^ Moreover, reduced organic molecules have larger electron ionization cross sections than more oxidized organics.^18–20^

Instead of theoretical estimation, studies have often relied on the assumption of universal sensitivity of EIMS to compounds within similar chemical classes. This assumption is based on studies that show evidence that the response factors (i.e., signals per unit mass of analyte) of EIMS are usually within 30% for analytes with the same functional groups^12^ and within ±50% for structurally different analytes.^21,22^ Notably, this level of uncertainty is similar in magnitude to the variability of FID response when no structural information is known, suggesting it is reasonable to similarly consider EIMS as a nearly universal detector, which would provide near-universal quantification without sacrificing chemical resolution. However, these estimates are based on relatively small numbers of commercially available standards used to build calibration curves in varied literature. Consequently, no robust estimate is available for the uncertainty introduced when using this assumption for analysis of the types of complex oxygenated products typically present in environmental mixtures, though this assumption is commonly implicitly or explicitly used when analyzing samples with many unidentified peaks.

The goal of this work is to quantify the extent to which EIMS can be applied as a near-universal detector within a given chemical class and the uncertainty introduced in doing so. In particular, the goal is to quantify uncertainty for the types of compounds for which standards are typically not available - the degradation products formed through oxidation reactions, particularly in secondary organic aerosols (SOA). Our approach is to compare and correlate FID and EIMS signals for samples of laboratory-generated atmospheric aerosols with the assumption that FID sensitivity is roughly universal per unit carbon mass. The individual species for comparison are preseparated from the complex secondary organic aerosol mixture with a GC. This work is intended to improve the community’s ability to comprehensively quantify analytes in environmental samples by characterizing the uncertainty in existing approaches and extending them beyond the limited range of commercially available analytes of interest. This result is achieved using a systematic approach to examine the treatment of EIMS as a near universal detector for chemically similar compound classes. An FID response is determined across the oxidation products based on empirical parametrizations of their individual measured molecular compositions.^5^ Comparing these data indicates that using EIMS as a near-universal detector introduces less than 25% uncertainty in most cases.

## Experimental Methods

### Instrument Configuration

Laboratory-generated atmospheric aerosols (SOA) were sampled by a Thermal desorption Aerosol Gas chromatograph (TAG), with two detectors: a time-of-flight EIMS (Aerodyne Research Inc., mass resolution *m*/Δ*m* = 4000)^23^ and an FID (Agilent Technologies, Inc.). The TAG-EIMS/FID enables online analysis of airborne particle-phase oxygenated organics through sample collection by impaction followed by separation of isomers by GC. The ionization energy of the electrons in the EIMS was 70 eV. Details of the instrumentation are described elsewhere.^24^ In brief, the TAG collects aerosol samples by impaction into a passivated steel cell at a sample flow rate of 9 standard liters per minute (slpm),^25^ typically for 5–15 min in this work with an equivalent volume of 0.045–0.135 m^3^ air. Liquid chemical standards are injected into the cell through the automated liquid injection system of the TAG.^26^ The samples collected by the cell are then transferred to the GC column through programmed thermal desorption through a heated (300 °C) valveless interface manifold that isolates sampling from the analytical system.^27^ A polar GC column (MXT-WAX, 17 m × 0.25 mm × 0.25 μm, Restek) wrapped on a temperature-controlled metal hub is used for the separation of oxygenated organic compounds (50 to 250 °C at a rate of 10 °C/min and then held for 25 min). The TAG cell was heated to 300 °C for the sample desorption at the heating rate of 50 °C/min and a flow rate of 1 standard cubic centimeter per minute (sccm) of helium gas was used as the carrier flow during the GC analysis. Though analysis of oxygenates by GC (in general) or TAG (specifically) typically relies on derivatization to convert difficult-to-elute polar functional groups into easier-to-elute groups,^28^ this approach is not employed here to minimize chemical alterations to the functionality of the analytes reaching the detectors. The GC column effluent is split to the two detectors, 0.7 sccm to EIMS and 0.3 sccm to FID, using a heated and passivated tee together with heated fused-silica transfer lines for simultaneous measurements by EIMS (0.17 m at 225 °C) and FID (0.5 m at 300 °C).^24^ The FID temperature was maintained at 300 °C. Hydrogen and zero air flows to the FID were at 40 and 400 sccm, respectively. The EIMS ionizer temperature was at 280 °C, with the ionization energy of the electrons at 70 eV and a mass range up to m/Q = 600 Th. An Iodide-Chemical Ionization Mass Spectrometer (I-CIMS) was used in separate experiments to identify molecular formulas associated with different peaks using methyl iodide to produce iodide ions; more details of the I-CIMS and the experimental setup can be found elsewhere.^24^

### Samples

The TAG-EIMS/FID was used to analyze particle-phase oxygenates generated through simulated atmospheric oxidation. Aerosols were generated through gas-phase O_3_ and/or OH oxidation of limonene, 1,3,5-trimethylbenzene (TMB), and eucalyptol using a Potential Aerosol Mass (PAM) oxidation flow reactor (OFR).^29^ For the convenience of the discussions later, a given set of oxidation experiments is discussed as “precursor-oxidant” (e.g., limonene-O_3_). Experiments were conducted at 25 °C, 40–50% relative humidity, and a constant gas flow rate of 12 L min^–1^ through the OFR. Precursors were injected into a carrier gas of synthetic air through use of an automated syringe pump at liquid flow rates ranging from 0.95 to 1.9 μL h^–1^ (mixing ratios of 236 to 472 ppbv). During ozonsolysis experiments, 35 ppmv O_3_ was injected at the OFR inlet. During photooxidation experiments, OH and HO_2_ were generated via O_2_ + H_2_O photolysis at 254 and 185 nm; over the range of conditions that were used, the estimated OH exposures in the OFR were in the range of 4× 10^10^ to 7× 10^11^ molecules cm^–3^ s.^30^ These experiments are used here to generate mixtures representative of atmospheric aerosols, but are not used to probe specific chemical questions, so further details of each experiment are not provided here. Bi et al.^31^ have previously demonstrated that products formed and analyzed in these experiments contain 5–10 carbon atoms and 1–5 oxygen atoms with molecular weights mostly between 130 and 200 g/mol, determined using detection by a chemical ionization mass spectrometer.

In addition to oxidation products, liquid chemical standards, including 1,12-dodecanediol, vanillin, and deuterated-alkanes (C_16_, C_20_, C_24_, and C_26_, C/D/N Isotopes, 98% purity) are injected into the sample collection cell through the automated liquid injector on TAG. All nonlabeled standards were purchased from Sigma-Aldrich at 97–99% purity.

### Comparison between Detectors

Each sample generates two chromatograms (EIMS and FID), which have minor differences in retention times due to differences in transfer line lengths. The retention time of the FID chromatogram is linearly corrected to align with EIMS by matching the retention times of the five most abundant shared peaks. EIMS and FID are compared to quantify the extent to which EIMS sensitivity correlates with FID sensitivity, which is known to be roughly universal. Although the sensitivity of FID may be variable based on the oxygen content, nearly all analytes are moderately oxygenated for the set of experiments performed, so this correction is expected to introduce only minor variability. For a subset of peaks with known molecular formulas, the O/C-based corrections for FID sensitivity were estimated to be a factor of 0.74 ± 0.06 (i.e., FID responses to the studied products were ∼74% compared to the response to *n*-alkanes and varied on average by only ∼10% between compounds) (Table S1). Molecular formulas associated with a total of 35 peaks were identified using an Iodide Chemical Ionization Mass Spectrometer (I-CIMS)^24^ in a separate series of experiments using the same precursor VOCs and oxidants. For simplicity of comparison between FID and EIMS, the four set of experiments discussed here did not have an I-CIMS connected to the same GC effluent. Our approach is therefore to focus on quantifying the variability of EIMS sensitivity relative to FID sensitivity, based on the underlying assumption that FID sensitivity is relatively stable and approximately universal (within 10% between all 35 compounds, as described).

Comparison was performed both “peak-by-peak,” i.e., integrations of FID and EIMS peaks compared (sum of all *m*/*Q* signals for one peak for EIMS reported as ions × seconds/extraction), and “point-by-point”, i.e., direct correlations of the FID chromatogram to the total EIMS chromatogram (summation of all measured mass spectral ions, or “total ion chromatogram” i.e., TIC). Direct point-by-point comparison provides a comprehensive way to compare detectors, even for peaks that are too poorly resolved to be integrated. However, this approach may be subject to biases or errors due to any uncorrected differences in retention times, differences in peak shapes, differences in baselines and backgrounds, or the presence of contaminants in only one detector or transfer line. This approach is supplemented by peak-by-peak comparisons of integrated peak areas in each chromatogram, which is more representative of the typical approach to quantifying chromatographic analytes but is limited to well-resolved analytes.

Since the signal units are not the same between EIMS and FID, for each point or peak, we compare the measured EIMS/FID ratio to the average EIMS/FID slope and use the relative difference to quantify the variability between EIMS and FID responses. So, the relative residual is calculated as

1 $${Relative\;Residual}_{i}=\frac{\left|S_{measured,i}^{EIMS/FID}-S_{fitted,i}^{EIMS/FID}\right|}{S_{fitted,i}^{EIMS/FID}}$$

where *S*_measured,*i*_^EIMS/FID^ and *S*_fitted,*i*_^EIMS/FID^ are the measured EIMS/FID signal and the linearly fitted EIMS/FID slope. Data analysis was performed with the TERN software package^32^ in the Igor Pro (Wavemetrics, Inc.) programming environments.

### Peak Integration

Instead of integrating EIMS data using a single mass spectral ion, as is typical, the total ion signal is integrated to more accurately represent the full amount of signal generated and provide a more direct comparison to the FID. However, this approach limits analysis to peaks that can be reasonably well resolved in both EIMS and FID. This requirement is met by selecting only chromatographic peaks which have peak height larger than 3% of the most abundant peak in each EIMS chromatogram. Peaks are integrated based on their full width at half-maximum (fwhm), determined by fitting peaks to an idealized Gaussian shape. Peak area is determined as total signal in the range [retention time – fwhm, retention time + fwhm], which is expected to capture approximately 98% of the total peak area and is not strongly sensitive to differences in detector properties such as noise or signal-to-noise or peak shape, which can strongly influence peak fitting for integration. The values of fwhm were similar but not the same for peaks from both the detectors due to differences in detector flow characteristics and transfer lines, so this approach seeks to directly compare the same region of the chromatogram. While direct integration of fitted peaks using a more realistic peak shape (e.g., exponentially modified Gaussian) similarly achieves peak-to-peak comparison, such an approach carries uncertainties in the peak fitting, which might more strongly depend on e.g., noise in the data, peak shape, or differing sensitivities of coeluting peaks. The direct comparison approach implies an assumption that the retention time (RT) between EIMS and FID is well-aligned, and the peak shape of the same compound is identical across the two detectors; though usually true, a few exceptions to this assumption will be examined in detail in the discussion. Retention time alignment reduces the risk of misidentifying an analyte peak between the two detectors, in case their sensitivities differ significantly to the same analyte. These criteria provided 36, 28, 39, and 26 chromatographic peaks for limonene-O3, limonene–OH, TMB–OH, and eucalyptol–OH, respectively, for which both EIMS and FID peak areas were quantified.

## Results and Discussion

### Point-by-point Comparison of Chromatograms

The total EIMS chromatograms are generally almost identical to the FID in all samples (Figure 1). The relative residual between detectors is lower than 0.2 (i.e., 20%) most of the time, though there are some exceptions observed for high abundance peaks, particularly in eucalyptol–OH experiment. Mean relative residuals are 0.10, 0.04, 0.07, and 0.07 for limonene-O_3_, limonene–OH, TMB–OH, and eucalyptol–OH, respectively (Table 1). In other words, total EIMS signal typically varies from FID signal by only 5–10% on average, and almost always by less than 20%, for the oxidation products examined in this study. The tight similarities between FID and EIMS is clear when examined as a direct correlation Figure 2, with a linear correlation coefficient of *R* ^2^ = 0.86. Furthermore, some outliers (e.g., observed as mismatched peaks in Figure 1 and loops falling off the correlation in Figure 2) are due in part to differences in peak shape in the detectors as opposed to true deviation. As shown in Figure S1, for a small number of analytes the peak shape may not be the same, probably due to the difference in transfer line length and temperature. Other outliers may represent true deviation between FID and EIMS sensitivity. For example, Peak 1, 2, and 3 in Figure 1d are liquid standards of perdeuterated alkanes injected into the sampling cell. The perdeuterated alkanes have similar peak shapes between EIMS and FID yet still show roughly a factor of 2 difference in peak height (or about 30% higher EIMS/FID peak area ratio), in contrast to most other peaks, which are oxidation products, consistent with previous studies.^18–20^ These differences are discussed in more detail below and may indicate the utility of EIMS as a universal detector within a compound class, but higher uncertainty between compound classes, as has been observed for FID.

**Figure 1:**
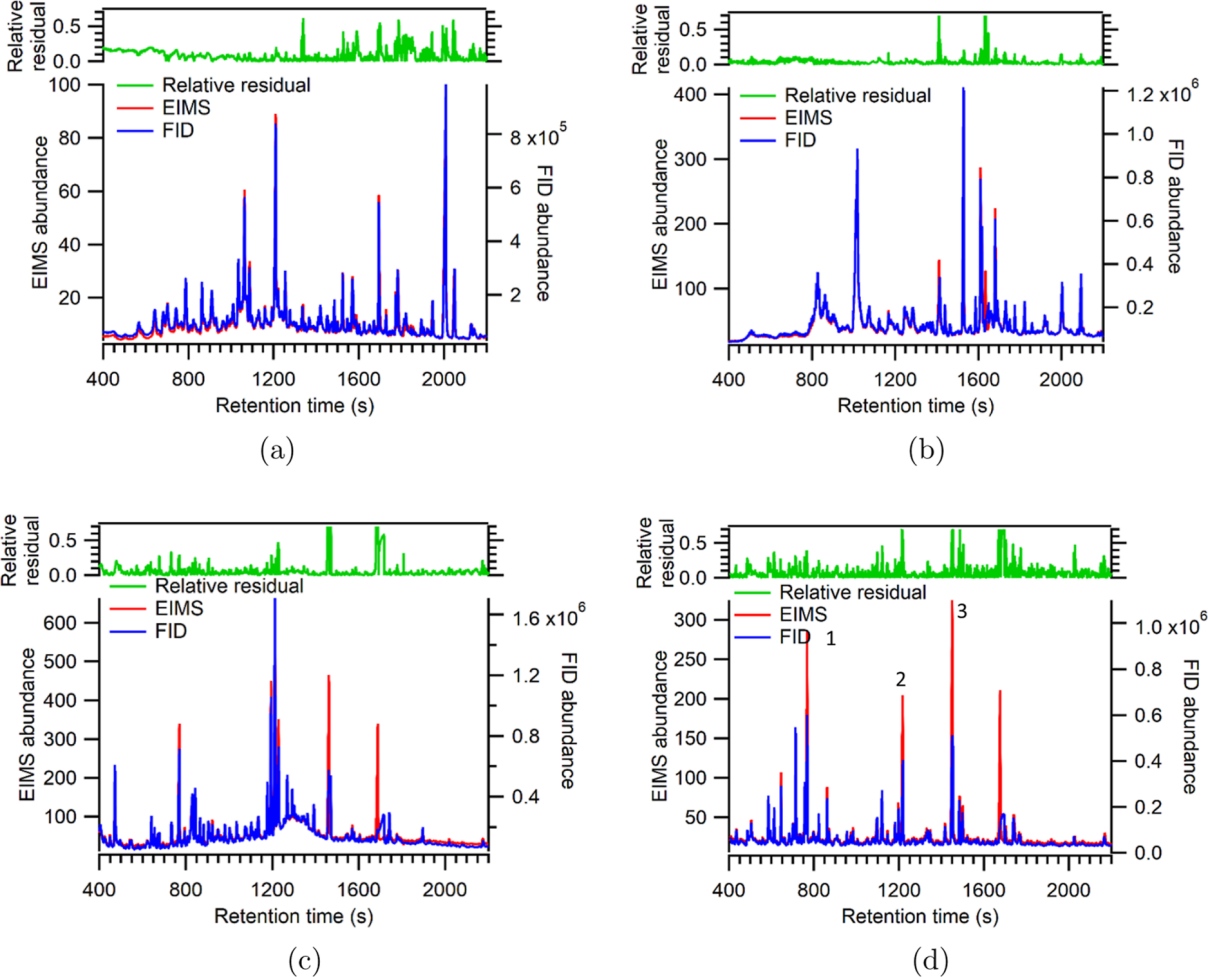
Overlay of FID and EIMS chromatograms of a sample in the (a) limonene-O_3_, (b) limonene–OH, (c) TMB–OH, and (d) eucalyptol–OH experiment. The top figures show the time-series of relative residual between FID and EIMS. A zoomed-in view of Figure 1a is available in Supporting Information showing aligned retention times (Figure S2). Relative scaling of FID and EIMS are determined by the average slope of their linear correlation (shown in Figure 2).

**Table 1:** Mean Relative Residual and Relative Standard Deviation of EIMS/FID Response in Oxidation Experiments

oxidation experiments	mean point-by-point relative residual (±standard deviation)	mean peak-by-peak relative residual (±standard deviation)
limonene-O3	0.10 ± 0.09	0.15 ± 0.20
limonene–OH	0.04 ± 0.10	0.22 ± 0.25
TMB–OH	0.07 ± 0.22	0.28 ± 0.41
eucalyptol–OH	0.07 ± 0.21	0.19 ± 0.11

**Figure 2:**
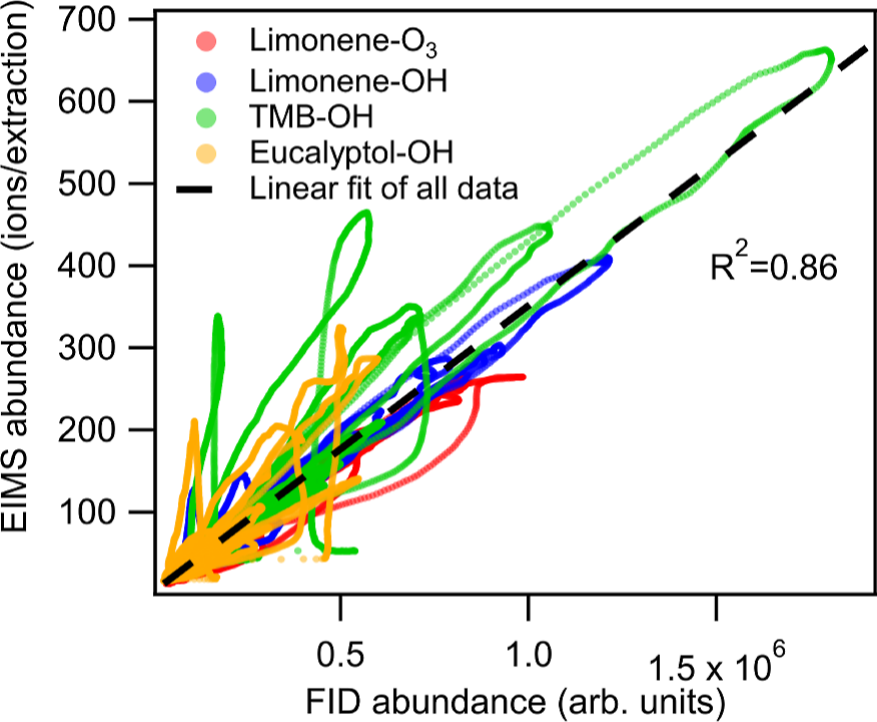
Correlation of point-by-point signals between EIMS and FID. Dashed line is the linear fit of all data points with a *R* ^2^ = 0.86. (Note: FID abundance values are provided by the instrument without any external calibration. Magnitude of values cannot be compared directly to infer sensitivity of the instruments).

### Peak-By-Peak Comparison of Chromatograms

While a point-by-point comparison provides a more comprehensive approach to comparison, it is subject to shifts in peak shapes and other nonidealities. In contrast, a comparison of actually integrated peaks demonstrates an tight linear correlation between EIMS and FID peak areas (*R* ^2^ = 0.98) of all 129 oxidation products (Figure 3). The relative residuals for EIMS peak areas from this fit are 0.23 ± 0.29, meaning that EIMS sensitivity can be predicted from FID sensitivity to within 23%. In particular, while the oxygenated injected standard 1,12-dodecanediol agrees well with this fit, deuterated alkanes show up to 50% higher signal in the EIMS. We therefore conclude that an EIMS can be comparable to an FID in its near-universality within a compound class and that an introduced oxygenated standard provides a good estimate of EIMS sensitivity for oxygenates within complex environmental (or at least atmospheric) mixtures, but that the same calibration applied across compound classes may introduce more error.

**Figure 3:**
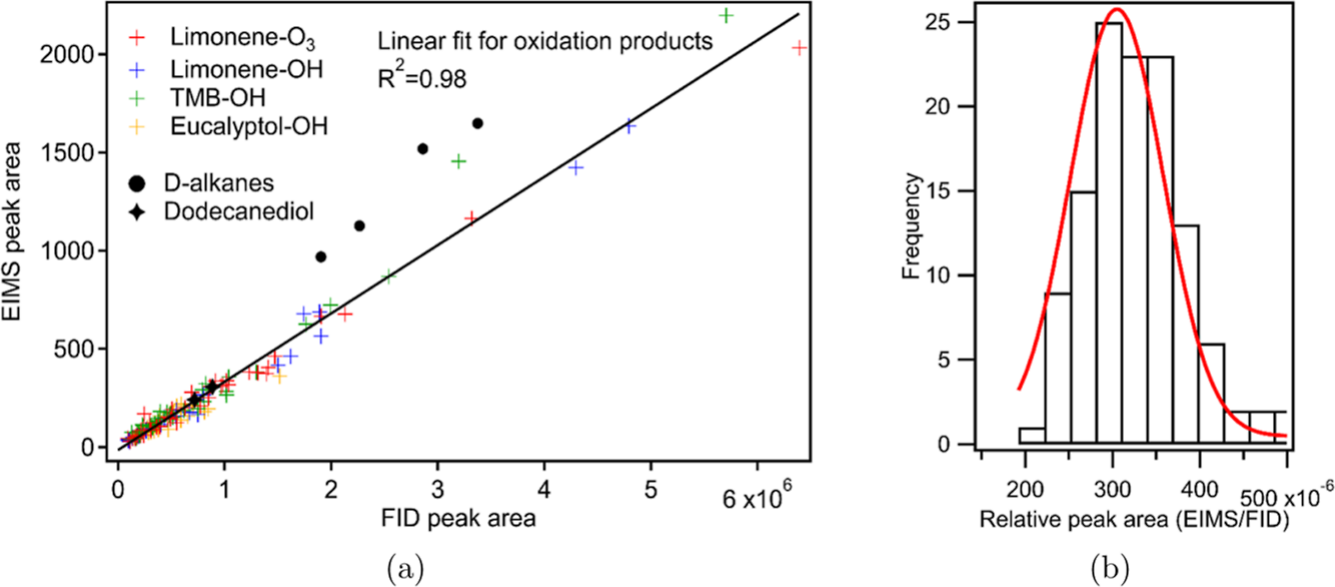
(a) The correlation between EIMS and FID peak area by peak-by-peak approach. A zoomed-in image is available in Supporting Information (Figure S3). (b) Histogram of relative peak area between EIMS and FID with fitted Gaussian distribution. Mean EIMS/FID peak area based on the guassian fit was 305 × 10^–6^ and the standard deviation was ∼17.5% of the mean EIMS/FID peak area (i.e., 53 × 10^–6^).

In addition to the relative residual, the variability in the EIMS sensitivity can be quantified as the deviation of the EIMS/FID ratio from the average ratio, again assuming that the FID response factors are relatively universal responses for these analytes. The distribution of the ratios (Figure 3b) demonstrates a relatively normal distribution with an overall relative standard deviation of 21%. The relative residuals of each oxidation system are 15%, 22%, 28%, and 19% for compounds in limonene-O_3_, limonene–OH, TMB–OH, and eucalyptol–OH experiments, respectively (Table 1). Therefore, a peak-by-peak comparison further supports an estimated variability of 20–30% for EIMS response factors of individual atmospherically relevant oxygenates present in SOA.

Although the presence of oxygenated functional groups likely impacts both EIMS and FID sensitivity, and the degree of such impacts may not necessarily be the same as those for FID sensitivity, this study shows that empirical linear correlations between EIMS and FID sensitivity exist at least across a relatively diverse set of oxygenated compounds. The similarity between FID and EIMS responses may at first glance be surprising, as EIMS is expected to have a molar response as it relies on collision between electrons and individual molecules, while FID is known to be proportional to the mass of carbon entering the detector.^5,15^ While the theoretical representations of the ionization cross-section are complex,^11^ they have been found to be linearly correlated with carbon atom numbers within a given structurally similar class of compounds (e.g., alkanes or alkylbenzenes).^14^ Consequently, ionization cross-section scales with molecule size and thus molecular weights for a given class of compounds. EIMS response can therefore be estimated as a molar response scaled by the molecular weight, i.e., the mass of the analyte, and thus considered as a mass-dependent detector.

While there is high linearity between EIMS and the FID peak area for particle-phase oxidation products and injected 1,12-dodecanediol, deuterated alkanes (round markers in Figure 3a) exhibit a somewhat different linear relationship. Given that the sensitivity of an FID to oxygenates deviates from that of hydrocarbons,^5^ observed differences in the EIMS/FID ratio may be due in part to deviations from the assumed near-universal response of the FID. However, this alone does not explain the observed differences in the slopes of oxidation products and d-alkanes. Instead, differences in the ratios may be due in part to the impact of other molecular parameters, such as polarizability or shape, on the ionization cross section, which are not expected to have a large impact on the FID response. This difference supports previous observations that calibration using chemically nonsimilar compounds yields larger uncertainty. The deuterated alkanes here support prior estimates that calibration across substantially different compounds yields ∼50% uncertainty, but there is not enough data in this work to confidently quantify uncertainty under such conditions. Instead, this work focuses on uncertainty across broadly similar compounds, since some degree of chemical similarity is typically achievable using commercially available standards.

Generally, it seems that the different impacts of molecular parameters in EIMS and FID cause the response factors to differ by up to ∼50% between widely disparate compound classes (e.g., C_16_–C_26_ hydrocarbons and C_5_–C_10_ oxygenates). However, within a broadly defined chemical class, EIMS response factors vary by only 20% and a near-universal response factor can be estimated using injected standards from within that class. This finding is consistent with smaller previous studies that estimated an uncertainty of 30% for atmospheric organic samples based on the introduced standards.^21,22^

## Conclusions

We examine the correlations between FID and EIMS through simultaneous FID and EIMS measurements of particle-phase organics in laboratory-generated secondary organic aerosol. The nearly identical chromatograms from the two detectors show that the signals of FID and EIMS are highly correlated for atmospherically relevant samples (SOA studied in this case). Such correlation is further strengthened by the linear relationship between FID and EIMS peak area of all compounds identified in the oxidation experiments, which is found to vary by less than 25%. Compounds of significantly different functional groups may differ in their response factors by up to 50%. The findings demonstrate that, as long as some context about a sample is available with which to broadly categorize unknown analytes (e.g., type of functional groups and/or physicochemical properties expected based on where the sample was taken from), EIMS can be used as a near-universal detector similarly to an FID and can be calibrated using authentic standards with broadly similar properties. We note that in any given system, sensitivity may be nonuniversal due to operational effects such as transfer efficiency from the sample to the GC, inefficient transfer through the GC, or other upstream effects that may require the introduction of additional calibrants.^28^ Nevertheless, a shared response factor across chemically similar compounds suggests that complex mixtures can be reasonably calibrated by only a small number of related analytes, resulting in moderate uncertainty (less than 25% in most cases).

We note that the compounds examined in the study may not cover the entire range of chemical species produced in atmospheric reactions, let alone present in a broader suite of environmental samples, because oxidation experiments were performed with a limited number of precursors and oxidants. Furthermore, the range of chemical properties of the analytes studied is limited by the collection method (the collection cell used samples only particle-phase components) and the analytical method (low volatility and/or high polarity elute poorly through a GC column). Consequently, the relatively narrow range of molecular weights could generate less variability in ionization cross sections, reducing the variability of EIMS response factors and thus increasing the apparent universality. Nevertheless, the data presented here provide a quantitative estimate of the uncertainty associated with treating EIMS as a universal detector within a reasonably known context and broad chemical similarity. Given that quantification of unknowns in complex environmental EIMS samples already frequently relies on an assumption of similar response factors between chemically related compounds,^21,22,33,34^ this work enhances understanding of the constraints and uncertainty of such an approach.

## Supplementary Material



## Notes

Discussion of the calculation of the FID response factor and figures showing differences in EIMS and FID peak shapes, retention time alignment, and zoomed-in view of EIMS peak area (PDF)

## ∥

Division of Chemistry and Chemical Engineering, California Institute of Technology, Pasadena, CA-91125, United States
