# Supplementary material for: Variability in the Universality of Electron Ionization Mass Spectrometry Response to Oxygenates in Complex Environmental Mixtures
Source: Anal Chem. 2025 Dec 8;97(50):27537–43. doi: 10.1021/acs.analchem.5c04153 (PMC12750400; doi:10.1021/acs.analchem.5c04153)
Supplement: Supplementary file 1 [file ac5c04153_si_002.pdf]

Supporting Information: Variability in the Universality of Electron Ionization Mass Spectrometry Response to Oxygenates in Complex Environmental Mixtures

Purushottam Kumar,[†,§] Chenyang Bi,[‡,§] Brian Lerner,[‡] John Jayne,[‡] Manjula R. Canagaratna,[‡] and Gabriel Isaacman-VanWertz[*,†]

†*Department of Civil and Environmental Engineering, Virginia Tech, Blacksburg, VA-24061, United States*

‡*Aerodyne Research Inc, Billerica, MA-01821, United States*

¶*Present Address: Division of Chemistry and Chemical Engineering, California Institute of Technology, Pasadena, CA-91125, United States*

§*P.K. and C.B. contributed equally to this work.*

E-mail: ivw@vt.edu

Table of Contents

1. Calculation of correction factor S3

2. Table S1: List of 35 peaks used in the calculation of correction factor S4

3. Figure S1: Example of mismatches in peak shape between EIMS and FID S5

4. Figure S2: Zoom in view of aligned retention times in Figure 1a. S5

5. Figure S3: Zoom in view of EIMS peak area vs FID peak area from Figure 3a. ... S6

6. References S7

Calculation of correction factor

The elemental formula of a subset of flame ion detector (FID) peaks were identified using high-resolution time-of-flight chemical ionization mass spectrometry (HR-ToF-CIMS) for samples generated in the same oxidation experiments.[1] The formulae were used for correcting sensitivity response factors as described by Hurley et. al.[2] In brief, FID detection of hydrocarbons provides a near-universal response per unit carbon mass, which is easily obtained by a multi-point calibration to a hydrocarbon. The average response to carbon in oxygenates decreases proportionally to the oxygen-to-carbon ratio (O/C) of the compound. Though the exact decrease in response is driven by the chemical functional groups present, Hurley and co-workers have shown that per-carbon FID sensitivity can be estimated from O/C to within approximately 20% uncertainty for an individual analyte. We therefore calculate the mass or number of moles of an analyte from its FID peak area based on a calibration response factor to n-alkanes, with a correction for oxygenation based on the elemental formula identified by an I-CIMS (specifically, the FID response per carbon atom relative to n-alkanes = -0.54 O/C + 0.99, where O/C is the oxygen to carbon ratio in the target analyte).[2] The 35 identified peaks yield an average absolute FID peak area (arbitrary units) of 849753 with a standard deviation of 886783 and an average correction factor of 0.74 ± 0.06 (i.e., FID sensitivity for the studied oxygenates is a factor of $\sim$0.74 compared to the response to n-alkanes that have the maximum FID response i.e. 1). The list of all 35 compounds, their relative effective carbon number (rECN or effective carbon number over the number of carbons in that molecule, i.e. ECN / C) used in the calculation, and estimated ionization cross sections[3] is provided in Table S1. Ionization cross section is related to sensitivity in an EIMS and the average ionization cross section for these 35 compounds was 24.74×10^{-16} cm^2 with a standard deviation of 15.7%. This variability supports the uncertainty estimates of approximately 20% observed in this work.

Table S1: List of 35 peaks used in the calculation of correction factor for the FID response relative to FID response for n-alkanes and estimated ionization cross sections. Elememtal formulas were identified by CIMS.

S. No.	Chemical formula	rECN (or ECN/C)	Ionization cross section ($\times 10^{-16}$ cm^2)
Trimethylbenzene-OH			
1.	C6H10IO3	0.73	19.26
2.	C7H10IO3	0.77	20.69
3.	C9H12IO4	0.76	26.11
4.	C9H12IO5	0.7	27.21
5.	C8H12IO4	0.73	24.68
6.	C9H14IO5	0.7	28.67
7.	C9H14IO5	0.7	28.67
8.	C9H14IO4	0.76	27.57
9.	C9H14IO5	0.7	28.67
10.	C8H12IO4	0.73	24.68
11.	C9H12IO5	0.7	27.21
12.	C9H12IO4	0.76	26.11
13.	C9H12IO5	0.7	27.21
Eucalyptol-OH			
14.	C6H8IO3	0.73	17.80
15.	C6H10IO3	0.73	19.26
Limonene-OH			
16.	C7H10IO3	0.77	20.69
17.	C5H6IO4	0.57	16.01
18.	C9H14IO4	0.76	27.57
19.	C9H12IO4	0.76	26.11
20.	C8H12IO4	0.73	24.68
21.	C9H14IO4	0.76	27.57
22.	C9H12IO4	0.76	26.11
23.	C9H14IO4	0.76	27.57
24.	C9H14IO4	0.76	27.57
25.	C8H18IO4	0.73	29.06
Limonene-Ozone			
26.	C9H14IO3	0.82	26.47
27.	C7H10IO2	0.85	19.59
28.	C9H14IO3	0.82	26.47
29.	C9H14IO3	0.82	26.47
30.	C5H6IO4	0.57	16.01
31.	C10H14IO3	0.84	27.90
32.	C9H12IO4	0.76	26.11
33.	C9H16IO3	0.82	27.93
34.	C7H10IO4	0.69	21.79
35.	C7H8IO4	0.69	20.33
	Average ± st. dev.	0.74 ± 0.06	24.74 ± 3.87

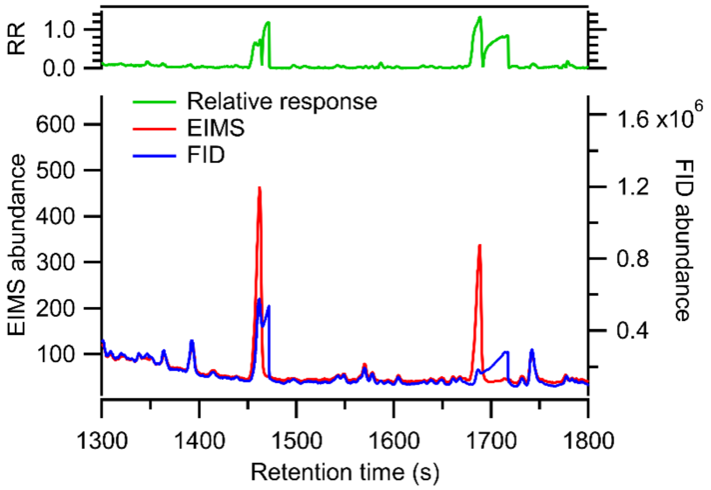

Figure S1: Example of mismatches in peak shape between EIMS and FID.

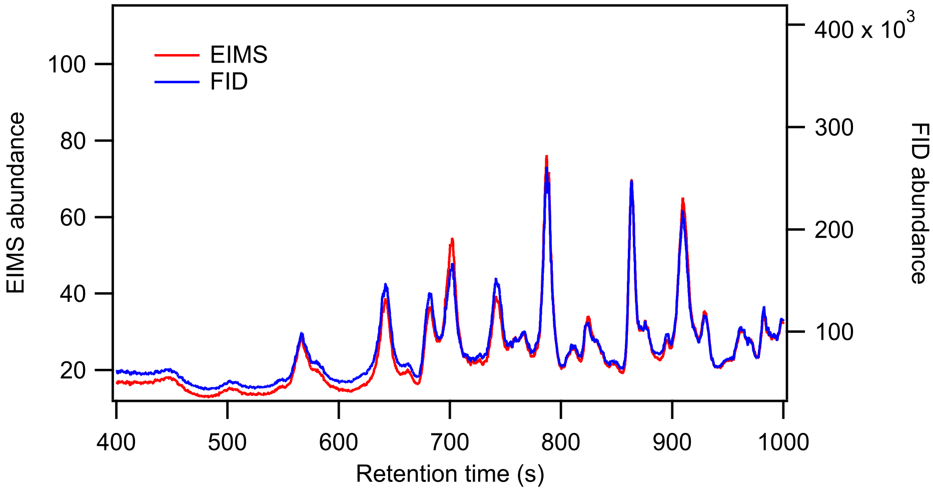

Figure S2: Zoom in view of aligned retention times in Figure 1a.

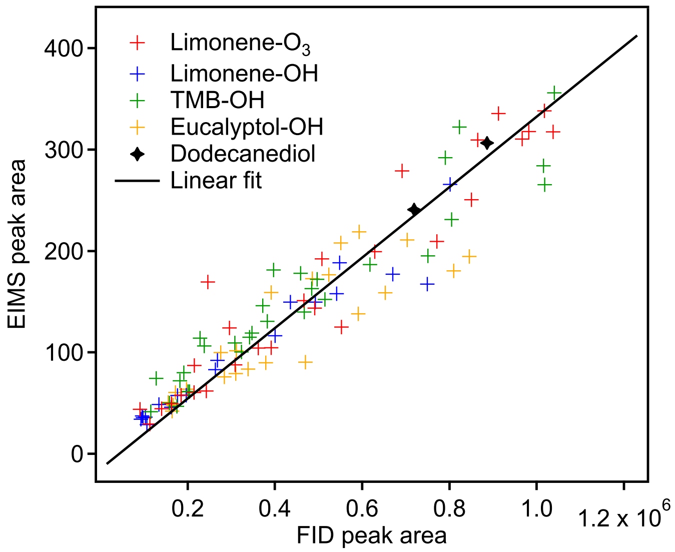

Figure S3: Zoom in view of EIMS peak area vs FID peak area from Figure 3a.

References

(1) Bi, C.; Krechmer, J. E.; Frazier, G. O.; Xu, W.; Lambe, A. T.; Claflin, M. S.; Lerner, B. M.; Jayne, J. T.; Worsnop, D. R.; Canagaratna, M. R.; Isaacman-VanWertz, G. Coupling a gas chromatograph simultaneously to a flame ionization detector and chemical ionization mass spectrometer for isomer-resolved measurements of particle-phase organic compounds. *Atmospheric Measurement Techniques* **2021**, *14*, 3895–3907.

(2) Hurley, J. F.; Kreisberg, N. M.; Stump, B.; Bi, C.; Kumar, P.; Hering, S. V.; Keady, P.; Isaacman-VanWertz, G. A new approach for measuring the carbon and oxygen content of atmospherically relevant compounds and mixtures. *Atmospheric Measurement Techniques* **2020**, *13*, 4911–4925.

(3) Fitch, W. L.; Sauter, A. D. Calculation of Retaltive Electron Impact Total Ionization Cross Sections for Organic Molecules. *Analytical Chemistry* **1983**, *55*, 832–835.
